# The SUPERFAMILY 2.0 database: a significant proteome update and a new webserver

**DOI:** 10.1093/nar/gky1130

**Published:** 2018-11-16

**Authors:** Arun Prasad Pandurangan, Jonathan Stahlhacke, Matt E Oates, Ben Smithers, Julian Gough

**Affiliations:** 1 MRC Laboratory of Molecular Biology, Hills Road, Cambridge CB2 2QH, UK; 2 Computer Science, University of Bristol, Bristol BS8 1UB, UK

## Abstract

Here, we present a major update to the SUPERFAMILY database and the webserver. We describe the addition of new SUPERFAMILY 2.0 profile HMM library containing a total of 27 623 HMMs. The database now includes *Superfamily* domain annotations for millions of protein sequences taken from the Universal Protein Recourse Knowledgebase (UniProtKB) and the National Center for Biotechnology Information (NCBI). This addition constitutes about 51 and 45 million distinct protein sequences obtained from UniProtKB and NCBI respectively. Currently, the database contains annotations for 63 244 and 102 151 complete genomes taken from UniProtKB and NCBI respectively. The current sequence collection and genome update is the biggest so far in the history of SUPERFAMILY updates. In order to the deal with the massive wealth of information, here we introduce a new SUPERFAMILY 2.0 webserver (http://supfam.org). Currently, the webserver mainly focuses on the search, retrieval and display of *Superfamily* annotation for the entire sequence and genome collection in the database.

## INTRODUCTION

SUPERFAMILY 1.75 (1) uses a library of 15 438 expert-curated profile hidden Markov models (HMMs) representing protein domains of known structure to predict the presence of structural domains in amino acid sequences. The domain sequences were obtained from the Structural Classification of Protein database (SCOP) (2). SCOP classifies protein domains into *Class, Fold, Superfamily* and *Family* level to understand structural, functional and evolutionary relationship between protein structural domains. The *Superfamily* level domains in SCOP share structural and functional properties that infer common evolutionary origin despite sharing low sequence identity. Whereas at the *Family* level, most homologous proteins cluster together with high sequence similarity suggesting clear evolutionary relationship and functional consistency (3). The SUPERFAMILY database provides domain annotations at both *Superfamily* and *Family* levels (4).

SUPERFAMILY provides various analysis tools to facilitate better analysis and interpretation of the database content. They include the identification of under- and overrepresentation of domains between genomes (5), construction of phylogenetic trees (6), analysis of the domain distribution of superfamilies and families across the tree of life (7) as well as providing ontology based annotations for SUPERFAMILY domains and architectures (8,9).

Here we present the development of new SUPERFAMILY 2.0 HMM library along with a major database update that includes the addition of SUPERFAMILY annotations for the all the protein sequences from the UniProtKB (10) and NCBI reference genome collections (11). We also introduce a newly developed webserver to mainly focus on the annotation of exponentially growing sequence data as well as to facilitate future integration with the SUPERFAMILY sister resources including dcGO (8) and D^2^P^2^ (12) to capture the combined information representing structure, disorder and domain centric ontologies in a single platform. In the following section, we discuss the development of new SUPERFAMILY 2.0 profile HMM library. Later, we discuss the annotation statistics for UniProtKB sequences and NCBI reference genome collection followed by the introduction of the new webserver and its basis functionalities. Finally, we discuss the future directions for the SUPERFAMILY resource.

## SUMMARY OF UPDATES

### SUPERFAMILY 2.0 profile HMM model library

In this update, we have created a new profile HMM library using sequences taken from the structural domain database SCOPe (13), CATH (14), ECOD (15) and PDB (16). Initially, we built the new HMMs for SCOPe domain sequences by filtering it at 95% sequence identity against the sequences present in the HMM library 1.75. The filtering and model building procedure was repeated for CATH and ECOD domain sequences followed by the full length protein chain sequences downloaded from PDB (16). For the purpose of building new HMMs, we used the HMMER package version 3.1b2 (17). For each new domain sequence, the program jackhmmer from the HMMER package was used to iteratively search for remote homologs to produce multiple sequence alignments (MSAs). The MSAs were used to generate HMMs using the Sequencing and Alignment Modeling Package version 3.5 (SAM) (18). The generated HMMs were converted to the HMMER 3.1b2 format. The number of iterative jackhmmer search steps was set to 5. The newly generated HMMs were carefully checked against each other and all models producing cross hits were removed. The new library contained 12,185 HMMs representing 10 668, 504, 279 and 734 models from SCOPe, CATH, ECOD and PDB sequences respectively. Finally, a new SUPERFAMILY 2.0 HMM library containing a total of 27,623 models was created by merging the new and existing 1.75 HMM library. Through the scop hierarchy page (http://supfam.org/scop), the user can browse full details of all the available domain sequences (including SCOPe, CATH, ECOD and PDB) used for building SUPERFAMILY 2.0 profile HMM library.

### UniProtKB sequence collection

Protein sequences were downloaded from the UniProtKB (ftp://ftp.ebi.ac.uk/pub/databases/uniprot/knowledgebase dated 29/03/2018) (10). It contained ∼112 million protein sequences classified into 63 244 complete genomes. The complete genomes represent 70%, 27%, 2% and 1% of Viruses, Bacteria, Eukaryotes and Archaea respectively. In UniProtKB, Viral genomes are most commonly found compared to Bacteria, Eukaryota and Archaea (Table 1). The SUPERFAMILY annotation pipeline was applied to the UniProtKB sequences. The annotation statistics show that 56%, 62%, 67% and 39% proteins were assigned with at least one *Superfamily* domain for Eukaryotes, Archaea, Bacteria and Viruses respectively (Table 1). The bacterial genomes have considerably higher percentage of *Superfamily* domain annotations compared to Viral genome. This is due to the fact that protein domain superfamilies in viruses do not have any structural and evolutionary relatives in modern cellular organisms and might be a source of new folds and functions (19). Overall, the complete proteome in UniProtKB contains 89 118 437 unique proteins and 64% have *Superfamily* domain annotations and 55% of amino acids have been mapped to *Superfamily* domains (Table 1).

**Table 1.** SUPERFAMILY annotation statistics for the UniProtKB and NCBI protein sequence collection

	No. of proteomes		No. of proteins		Proteins with assignments %		Amino acid coverage %	
	UniProtKB	NCBI	UniProtKB	NCBI	UniProtKB	NCBI	UniProtKB	NCBI
Eukaryota	1272	781	194 81 055	17 857 765	56	67	38	39
Archaea	793	671	2 136 652	1 822 967	62	63	59	60
Bacteria	17 277	93 480	66 475 668	346 500 943	67	67	62	64
Viruses	43 902	7194	1 025 062	303 337	39	21	39	31
Complete proteome	63 244	102 151	89 118 437	90 495 662	64	67	55	62

### NCBI complete genome collection

Protein sequences were downloaded from the NCBI Reference Sequence Database (ftp://ftp.ncbi.nlm.nih.gov/genomes/refseq dated 26/08/2017) (11). The genome collection has about 93 million protein sequences from 102 151 complete genomes. It contained 91%, 7%, 1% and 1% of Bacteria, Viruses, Eukaryotes and Archaea respectively with bacterial genomes being the most common (Table 1). Eukaryotes, Archaea, Bacteria and Viruses had 67%, 63%, 67% and 21% of proteins with at least one *Superfamily* domain annotations (Table 1). Overall, the complete proteome in NCBI contains 90495662 unique proteins and 67% have *Superfamily* domain annotations with 62% of amino acids have been mapped to *Superfamily* domains (Table 1).

After the major update, the SUPERFAMILY database contains 50 604 320 and 44 765 365 distinct protein sequences from UniProtKB and NCBI respectively. About 50% of the protein sequences (45 730 297) are common between UniProtKB and NCBI sequence collection. It is worth noting that the annotations for UniProtKB and NCBI sequences were performed using the SUPERFAMILY 1.75 HMM library.

### New webserver - SUPERFAMILY 2.0

The wealth of proteome sequence information continues to increase manifold with the recent advancement of sequence technologies. In order to meet the challenges involved in the analysis and interpretation of large proteome datasets, we have developed a new webserver (http://supfam.org). The webserver is built using a Perl based real-time web application framework called Mojolicious (https://mojolicious.org). In this new development, we have predominately focused on the search, retrieval and display of *Superfamily* domain annotations present in the database. We foresee the integration of some of the essential analysis and visualization tools into the new webserver that would eventually replace its predecessor in the near future. In the following section, we discuss some of the key features of the new webserver.

#### Genome browser

**Figure 1.**
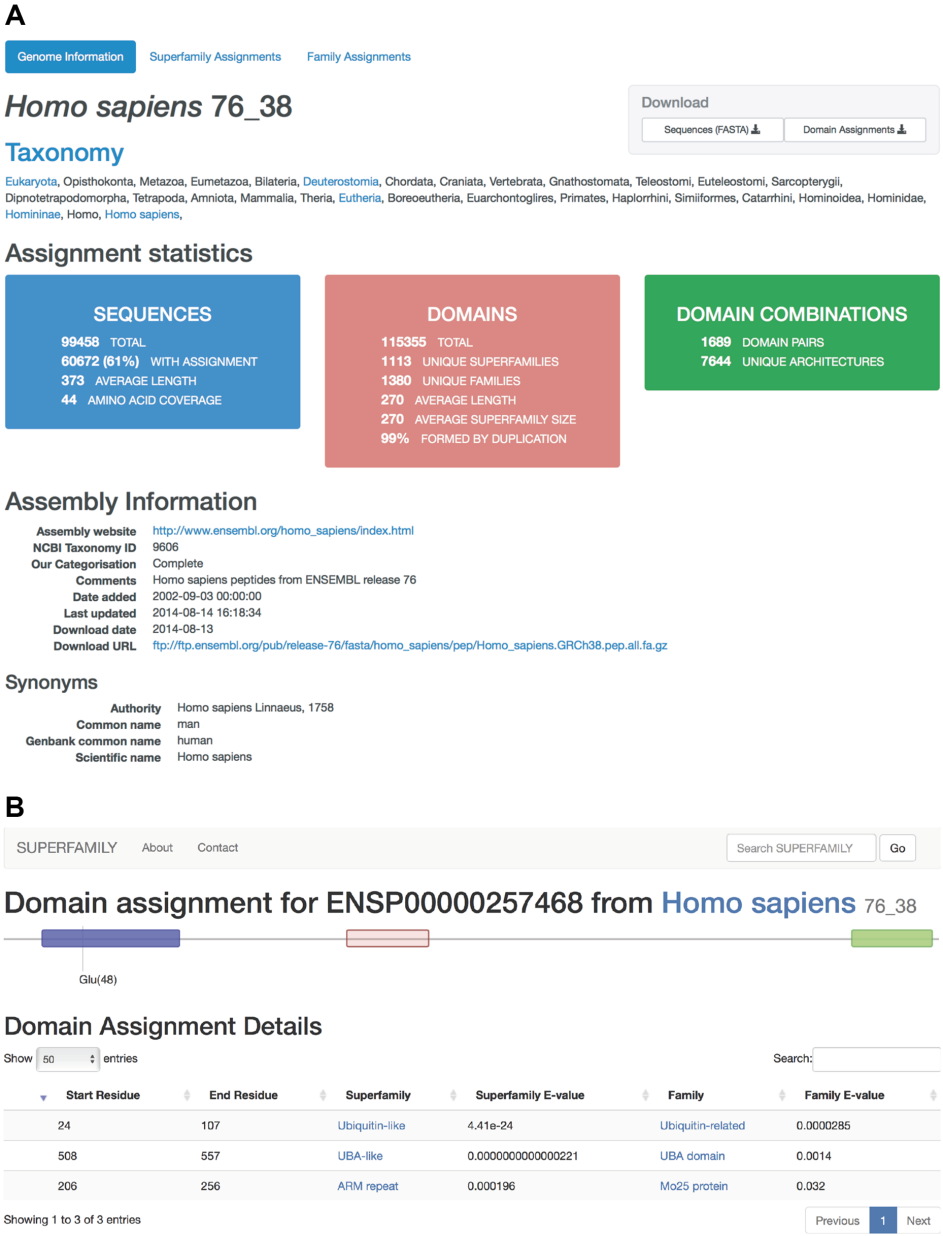
SUPERFAMILY webserver 2.0. (**A**) Genome summary page, showing SUPERFAMILY domain annotation statistics for Homo sapiens genome. The page also provide links to view and download SUPERFAMILY domain assignments. (**B**) Domain annotation page showing SUPERFAMILY domain predictions for the protein sequence id ENSP00000257468 of *Homo sapiens* genome.

The user can browse all genomes present in the database using the taxonomy tree of life (http://supfam.org/genome/hierarchy). For easy lookup and navigation, the webpage provides a navigation panel based on the taxonomic class information. Following the links, the user can reach the landing page of a genome that summarizes the annotations statistics (e.g. http://supfam.org/genome/hs) (Figure 1A). The genome summary page provides various annotations statistics including the percentage of sequences with *Superfamily* assignments, percentage of amino acid coverage, number of domains, superfamilies, families, domain pairs and unique architectures that represent predicted combination of *Superfamily* domains. Following the download links the user can download all protein sequences in fasta format along with its predicted *Superfamily* assignments as flat text files. The page also provides hyperlinks to access the list of all *Superfamily* and *Family* assignments found in the genome which intern provides access to the list of all protein sequences containing the predicted *Superfamily* and *Family* domains. A typical domain annotation page for a given protein sequence contains a list of regions on the protein sequence containing the predicted *Superfamily* and *Family* domains assignments along with their respective E-values and the closest structure derived from the family assignment procedure (4). The closest structure act as a potential template to build comparative models using SUPERFAMILY annotations (20,21). The domain annotation page also contains a graphical representation of the *Superfamily* domain organisation (shown as coloured blocks) laid on the sequence (shown as a straight line) (Figure 1B).

#### Genome statistics

For a quick look up, we provide a summary page listing all genomes present in the database along with the SUPERFAMILY annotations statistics as mentioned above (http://supfam.org/genome/stats). The genomes shown in the statistics page are organized into various model organisms (Eukaryotes, Prokaryotes and their respective strains), metagenome and pseudogenes. Most of the metagenomes annotated in the database were downloaded from the Joint Genome Institute as part of the environmental sequencing project (https://jgi.doe.gov). The pseudogenes are derived computational using in-house program from the Ensembl genome database (http://www.ensembl.org/). Various sequence collection including UniProtKB and NCBI are listed in a separate category called ‘Others’. Hyperlinks for the genome names provided in this page point directly to the respective genome summary page.

#### UniProtKB and NCBI genome collection

The majority of protein sequences annotated in the database are from UniprotKB and NCBI resources. These sequences are organised into a set of complete genomes that are available for viewing through the subgenome option (UniProtKB genomes: http://supfam.org/subgenome/up, NCBI genomes: http://supfam.org/subgenome/ncb).

#### Sequence and keyword search

The sequence search facility (http://supfam.org/sequence/search) allows the user to submit up to 1000 protein sequences and obtain its corresponding domain assignments based on the new SUPERFMAILY 2.0 HMM library. To avoid redundant computation and to speedup, the submitted sequences are searched against the SUPERFAMILY database for exact match with pre-defined domain assignments. Whenever a hit is not found, the sequences are searched against the SCOP domain sequences from ASTRAL (22) using BLAST (23). As a final stage with no hits, the sequences are searched against the SUPERFAMILY 2.0 model library using the HMMER package (17). In addition, the database can be searched using keywords that include protein sequence identifier, genome names, SCOP identifier and SUPERFAMILY model identifier.

## DISCUSSION

We are in the process of integrating the results from the disorder prediction using D^2^P^2^ (12) and domain centric ontologies using dcGO (8,9) with SUPERFAMILY domain prediction into a single platform in order to facilitate better interpretation of protein sequence, structure, disorder and function. As part of the Genome3D consortium, SUPERFAMILY domain predictions are used to build 3D structural models for proteome of various model organisms of significant importance (21). In line with that, the webserver will include new functionality to build 3D models on the fly based in the SUPERFAMILY domain predictions.
